# Cost-effectiveness of rapid, ICU-based, syndromic PCR in hospital-acquired pneumonia: analysis of the INHALE WP3 multi-centre RCT

**DOI:** 10.1186/s13054-025-05428-1

**Published:** 2025-08-08

**Authors:** Adam P. Wagner, Virve I. Enne, Vanya Gant, Susan Stirling, Julie A. Barber, David M. Livermore, David A. Turner, the INHALE WP3 study group, Adam P. Wagner, Virve I. Enne, Vanya Gant, Susan Stirling, Julie A. Barber, David M. Livermore, David A. Turner, Naseem Ahmed, Olugbenga Akinkugbe, Zoran Aman, Angela Aramburo, Georgia Bercades, David Brealey, Rhian Bull, Jane Cassidy, Jaime Carungcong, Benny P. Cherian, Antony Colles, Patricia Correia da Costa, Jeronimo Cuesta, Zaneeta Dhesi, Kerry Dresser, Matthew Dryden, Helder Filipe, Minnie Gellamucho, Ingrid Hass, Juliet High, Robert Horne, Hala Kandil, Michael Karlikowski, Nigel Klein, Kamal Liyanage, Damian Mack, Philip Milner, Sara Garcia Mingo, Daniel Martin, Luke Moore, Nabeela Mughal, Mark de Neef, Julie North, Justin O’Grady, Lauran O’Neill, Valerie Page, Alyssa Pandolfo, Robert Parker, Nehal Patel, Pooja Patel, Mark Peters, Giovanni Pipi, Karen Reid, Federico Ricciardi, Peter Riley, Charlotte Russell, Laura Shallcross, David Shaw, Suveer Singh, Ruan Simpson, Julian Sonksen, Sarah-Jane Stewart, Deborah Smyth, Ann Marie Swart, Jenny Tan, Carly Tooke, Laura Tous, Eleanor Tudtud, Ian Turner-Bone, Justin Wang, Victoria Waugh, Ingeborg D. Welters, Laura Wilding, Helen Winmill, Karen Williams, Xiaobei Zhao

**Affiliations:** 1 Norwich Medical School, University of East Anglia, Norwich Research Park, Norwich, NR4 7TJ UK; 2 National Institute for Health and Care Research Applied Research Collaboration East of England (NIHR ARC EoE), Cambridgeshire and Peterborough NHS Foundation Trust, Cambridge, UK; 3 Centre for Clinical Microbiology, University College London, London, UK; 4 Norwich Clinical Trials Unit, University of East Anglia, Norwich, UK; 5 Department of Statistical Science, University College London, London, UK; 6 Department of Microbiology, University College London Hospitals, London, UK; 7 Great Ormond Street Hospital, London, UK; 8 BUPA Cromwell Hospital, London, UK; 9 Royal Brompton and Harefield Hospitals, London, UK; 10 NIHR University College London Hospitals Biomedical Research Centre, London, UK; 11 Chelsea and Westminster Hospital NHS Foundation Trust, London, UK; 12 Birmingham Children’s Hospital NHS Foundation Trust, Birmingham, UK; 13 Barts Health NHS Trust, London, UK; 14 UK Health Security Agency, London, UK; 15 Royal Free Hospital, London, UK; 16 University Hospitals of North Midlands NHS Trust, Stoke, UK; 17 West Hertfordshire Teaching Hospitals NHS Trust, Watford, UK; 18 James Paget University Hospitals NHS Foundation Trust, Great Yarmouth, UK; 19 Royal Free London NHS Foundation Trust, London, UK; 20 University of Plymouth, Plymouth, UK; 21 London School of Economics, London, UK; 22 Liverpool University Hospitals NHS Foudation Trust, Liverpool, UK; 23 UCL Great Ormond Street Institute of Child Health, London, UK; 24 West Hertfordshire Teaching Hospitals NHS Trust, Watford, UK; 25 Dudley Group NHS Foundation Trust, Dudley, UK; 26 St George’s University Hospitals NHS Foundation Trust, London, UK; 27 Royal Liverpool University Hospital, Liverpool, UK; 28 Portsmouth Hospitals NHS Trust, Portsmouth, UK; 29 Cromwell Hospital, London, UK; 30 Aintree University Hospital, Liverpool, UK; 31 University of Liverpool, Liverpool, UK

**Keywords:** Hospital-acquired pneumonia (HAP), Ventilator-associated pneumonia (VAP), Molecular diagnostics, Syndromic PCR, Rapid PCR, Point-of-care, Antibiotic stewardship, Cost-effectiveness

## Abstract

**Background:**

Hospital-acquired and ventilator-associated pneumonia (HAP and VAP) are pneumonias arising > 48 h after admission or intubation respectively. Conventionally, HAP/VAP patients are given broad-spectrum empiric antibiotics at clinical diagnosis, refined after 48–72 h, once microbiology results become available. Molecular tests offer swifter results, *potentially* improving patient care. To investigate whether this potential is realisable, we conducted a pragmatic multi-centre RCT (‘INHALE WP3’) of rapid, syndromic polymerase chain reaction (PCR) in ICU HAP/VAP compared with standard of care. As the use of molecular tests impact on hospital resources, it is important to consider their potential value-for-money to make fully informed decisions. Consequently, INHALE WP3 included an economic evaluation, presented here. Its aim was to estimate the cost-effectiveness of an in-ICU PCR (bioMérieux BioFire FilmArray Pneumonia Panel) in HAP/VAP, informing whether to implement such technology in routine NHS care.

**Methods:**

We collected data on patient resource use and costs. These data were combined with INHALE WP3’s two primary outcome measures: antibiotic stewardship at 24 h and clinical cure at 14 days. Cost-effectiveness analyses were carried out using regression models adjusting for site. Sensitivity analyses explored assumptions and sub-group analyses explored differential impacts.

**Results:**

We found lower total ICU costs (including PCR costs) in the intervention (PCR-guided therapy) group. Average costs were £40,951 for standard of care compared with £33,149 for the intervention group, a difference of − £7,802 (95% CI: − £15,696, £92). For antibiotic stewardship, the PCR-guided therapy was both less costly and more effective than routine patient management. For clinical cure, we did not find PCR-guided therapy to be cost-effective due to fewer cases being cured in the intervention group.

**Conclusions:**

We found lower average ICU costs with the Pneumonia Panel. The pneumonia panel was cost-effective in terms of antibiotic stewardship, but not clinical cure.

*Trial registration*: Registered as ISRCTN16483855 on 5th August 2019.

**Supplementary Information:**

The online version contains supplementary material available at 10.1186/s13054-025-05428-1.

## Background

Hospital-acquired pneumonia (HAP) is defined as pneumonia arising > 48 h after admission [1, 2]. Ventilator-associated pneumonia (VAP) is pneumonia that arises over 48 h after endotracheal intubation [1]. These infections occur in between 5 and 40% of ICU patients [3, 4], leading to 10–50% increased mortality.

HAP and VAP can be caused by various bacteria, viruses, and fungi. Routine characterisation of these organisms takes 48 to 72 h. Until then, HAP/VAP patients are given broad-spectrum empirical antibiotics, refined once microbiology results become available [5]. Rapid multiplex polymerase chain reaction (PCR) tests can expedite this process, as well as offering improved sensitivity. In principle, these faster, more accurate, results might improve patient treatment and outcomes. They may also facilitate improved antibiotic stewardship. A growing literature indicates that these tests perform well in terms of identifying key pathogens and antibiotic resistance [6–10]. However, further evidence of their impact in clinical practice is required to inform implementation decisions.

The UK National Institute for Health and Care Excellence (NICE) consequently highlighted rapid testing in HAP as a research priority [11]. To address this topic, a pragmatic multi-centre randomised controlled trial (‘INHALE WP3’) was conducted [12, 13]. This investigated clinical outcomes and antibiotic stewardship of a rapid, syndromic, PCR test and targeted treatment for patients with HAP and VAP in intensive care units (ICUs).

If deployed based on clinical and or antibiotic stewardship benefits, rapid multiplex PCR tests would also require the commitment of financial resources–from the National Health Service (NHS) in the typical UK context. These include costs of the PCR-test equipment and consumables, along with the staff time spent conducting the tests. Given that the tests are not comprehensive (i.e. they do not seek all pathogens and resistances) these costs would be additional to routine microbiology. Changes in management of HAP/VAP patients may also impact on wider hospital resources and associated costs. It should be added that HAP and VAP themselves have substantial effects on the costs of providing care. For instance, patients receiving cardiac surgery who developed VAP were estimated to accrue additional costs for post-surgical recovery of £8,829 compared with cardiac patients without VAP (2013/2014 cost year; patients treated 2011–2014) [14]. Patients with traumatic brain injury who acquired HAP were found to spend 10.1 days longer in acute care [15]. A study of US Medicaid patients with non-ventilator hospital-acquired pneumonia between 2015 and 2019 found it was associated with an excess cost per case of $20,189 [16]. Hospital costs associated with HAP/VAP may be reduced if the infection can be better managed, with a shorter duration of ICU stay. More generally, improved stewardship–based upon swifter diagnostics–might reasonably be anticipated to reduce the accumulation of antibiotic resistance in the longer term, as well as reducing antibiotic consumption by individual patients. These factors may reduce long-term future costs.

Decisions regarding implementation of rapid multiplex PCR microbiological diagnostics require evidence around resource implications as well as effectiveness. Consequently, we conducted an economic analysis in parallel with the INHALE WP3 randomised clinical trial [12, 13]. This aimed to estimate the cost-effectiveness of the rapid, in-ICU syndromic PCR test evaluated in the trial, informing whether to implement it in routine clinical care.

## Methods

### INHALE WP3 clinical trial

The INHALE trial has been described in detail elsewhere [12, 13]. In brief, INHALE was an open-label RCT (randomised controlled trial) conducted across 14 ICUs (11 adult and 3 paediatric). Patients were eligible for inclusion if they were about to receive initial empiric antibiotic therapy for HAP or VAP. They were also eligible if they were about to have their HAP/VAP therapy changed for a worsening infection. As per standard practice, HAP and VAP were defined as pneumonia developing > 48 h after hospital admission or ventilation, respectively. Individuals were randomly allocated 1:1 to either ‘standard of care’ (control group, with empirical therapy and routine microbiology) or to rapid investigation with a rapid in-ICU, syndromic PCR (bioMérieux BioFire FilmArray Torch Pneumonia Panel; henceforth, the ‘Pneumonia Panel’) supported with a localised algorithm to translate the PCR results into antibiotic prescribing guidance [12]. The Pneumonia Panel was utilised in the ICU and results obtained with a median delay of 1.5 h (IQR: 1.4, 1.8) compared with a median of 73.7 h (IQR: 66.5,116.7) for routine microbiology [13]. Data were actively collected for each patient for 28 days.

Two co-primary outcomes were pre-specified [13]. First, superiority in antibiotic stewardship at 24 h post-randomisation, defined as the proportion of patients on active and proportionate antibiotic therapy within 24 h of clinical diagnosis, where ‘active’ is defined as receiving an antibiotic active against the organism(s) in vitro and ‘proportionate’ as not excessively broad-spectrum for the pathogen(s) identified. Second, non-inferiority in respect of clinical cure of pneumonia at 14 days post-randomisation. Cure was defined as the absence of: (i) death, where pneumonia was considered causative or contributory; (ii) septic shock, except when associated with a documented non-respiratory origin of infection; (iii) relapse of pneumonia; or (iv) other evidence that the original pneumonia was not cured. The target sample size was 552.

### Economic evaluation

To inform decisions on whether the Pneumonia Panel would represent good use of resources, specifically in the context of the UK NHS, we conducted two economic evaluations, based on the above two co-primary outcomes. For both, analysis was from an NHS secondary care perspective.

First, we considered the extra cost per additional person on active and proportionate antibiotic therapy within 24 h of clinical diagnosis (‘stewardship’). Secondly, we calculated the cost per additional clinical cure of pneumonia at 14 days post-randomisation (‘cure’). If the intervention group for either analysis was found to be both more costly and more effective than the control group, we then reported incremental cost-effectiveness ratios (ICERs), which show the extra costs per additional unit of effect produced.

### Costing methodology

Resources costed in the study included: days in ICU; non-ICU hospital admission costs; use of standard microbiology culture and susceptibility testing; use of the Pneumonia Panel in the intervention arm; prescribed antibiotics; and use of chest X-rays and CT scans. For ICU stays we had data from two sources. First, we obtained costs (and associated admission dates) for ICU and general hospital admission from hospital finance departments. These represented hospital income–a proxy for cost on the assumption that, within the NHS, there is no profit element–and covered the period from hospital admission to discharge, which could be considerably longer than the 28-day follow-up of the trial. For participants with multiple ICU stays, we focused on the ICU stay covering the contiguous period from INHALE randomisation to ICU discharge during this period. Details on costs for the whole hospital admission were less complete, as some patients were yet to be discharged from hospital at time of data extraction. Secondly, details of ICU stay were extracted from routine care records by trial research staff and entered on the study database. This extract included ICU admission and discharge dates, organ systems supported (adults) and critical care level (children). ICU data were collected from randomisation to day 21 or discharge, whichever was first. Such data cover a shorter period than hospital finance data and do not necessarily capture the total costs of ICU stays, which could extend beyond 21 days. Owing to this limitation, we prioritised ICU costs from finance departments, but where these were unavailable/incomplete, we utilised the censored trial collected data. Costs associated with stays beyond one-year were discounted at a rate of 3.5%.

We also collected data on other resource items that may be impacted by the addition of Pneumonia Panel to ICU care. In particular, the number of microbiological culture tests conducted was recorded in the study database and an estimate of the cost per test was obtained from the microbiology department of one participating hospital (£20 per test–expert opinion). This source also provided a cost estimate for routine in-house respiratory viral PCR (£40). Cost per test for the pneumonia panel were estimated using prices quoted by the manufacturer (bioMérieux), and included equipment costs, consumables, and quality control materials. We assumed one FilmArrayTorch instrument with two slots was purchased per ICU and estimated an annual equivalent cost with an operational life-time of five years, interest rate of 3.5%, and no re-sale value [17]. We also calculated costs based on leasing rather than outright purchase using costs available at time of analysis, this had very little impact on estimated cost per test. Costs were based on operation of the instrument at point-of-care inside the ICU (as in the RCT). We assumed five minutes of an NHS band 6 nurse conducting the test and estimated a total cost per test based on an estimated annual throughput of 360 tests per machine. Use of X-ray or CT scans was recorded from randomisation to either 21 days post randomisation, or to discharge/death, should this occur before 21 days. Use of these scans was costed using NHS reference costs, at £45 for a chest X-ray and £144 for a CT scan [18].

For costing antibiotics, research staff recorded details of all antibiotics prescribed and/or administered to patients from 7 days before enrolment to 21 days after. Recorded information included: antibiotic name; dose; treatment frequency; route (oral, intravenous, etc.); and start and end date and time of treatment. Identified drugs were costed using the online British National Formulary [19], and so related costs are limited to acquisition–they do not include other costs relating to administration, monitoring or antibiotic prescribing, which were not specifically captured.

In the base case analysis, costs are the sum of the individual patient costs during the contiguous ICU stay from randomisation, plus the cost of the Pneumonia Panel in the intervention arm. For a sensitivity analysis, we also consider ‘total’ costs: this comprises ICU and Pneumonia Panel costs (as in the base case) plus general hospital admission income such as ward stays. Costs related to the use of routine tests and antibiotics during the ICU admission are excluded to avoid double counting as they would have been included as components of the ICU costs. All costs identified were in 2020/21 UK pounds sterling.

### Outcomes used in the economic evaluation

INHALE WP3’s two outcome measures were used to inform two separate cost-effectiveness studies. These were (i) non-inferiority in clinical cure of pneumonia at 14 days post-randomisation and (ii) superiority in antibiotic stewardship at 24 h post randomisation [12, 13].

### Analysis

Usage and costs for each resource category are compared using unadjusted differences in means with 95% CIs. Base case analysis estimated costs from the perspective of the hospital ICU and adopted an intention to treat (ITT) approach, i.e., participants were analysed in the group to which they were allocated. Regression models (at minimum adjusting for site using a random intercept) were fitted to estimate treatment effects comparing the intervention (Pneumonia Panel) and control arms for costs (linear mixed models) and each co-primary outcome (logistic mixed models). We report both odds ratios (ORs) and corresponding proportional differences for the effectiveness outcomes–the latter are used in the economic evaluation/cost-effectiveness (CE) planes. Confidence intervals (CIs) for adjusted effectiveness and cost estimates, and wider explorations of uncertainty using CE planes and cost-effectiveness acceptability curves, were obtained using non-parametric bootstrapping (resampling 1,000 times).

To explore the impact of uncertainty in adopted assumptions, we conducted multiple sensitivity analyses. These included: (i) base case analysis but only including participants for whom hospital finance data were available; (ii) base case analysis truncated to 14 days post randomisation; (iii) base case excluding ICU stays > £200,000 (i.e. treating such stays as outliers); (iv) base case adjusted for baseline covid status, SOFA/pSOFA score (combined as in the main clinical paper[13]), and other infection; and (v) analysis using ‘total’ hospital costs (see costing section for definition). Further, we reviewed the base case results for specified subgroups by adding to the model interaction terms between trial arm and a subgroup indicator for: adults versus children; Covid-19 status at randomisation; HAP versus VAP.

Methods used in these economic analyses, conducted alongside the RCT, are described in detail in the health economic analysis plan (HEAP). This was written prior to the health economic analysis being conducted and was made available on the INHALE website hosted by the Norwich Clinical Trials Unit (https://norwichcrtu.uea.ac.uk/ctudocs_public/inhale/heap_1_4.pdf). As part of the submission process a CHEERS checklist for reporting economic evaluations was completed and the current work adheres to relevant items.

INHALE study ethical approval was from the London-Brighton and Sussex Research Ethics Committee (19/LO/0400). Trial registration: ISRCTN16483855.

## Results

### Participants

In line with the clinical analysis [13], we had 531 (263 control and 268 intervention) and 533 (265 control and 268 intervention) observations for stewardship and clinical cure respectively. Among patients with one or both of these outcomes (n = 542), finance department data on ICU resource use was available for 468 (226 control and 242 intervention); this information was augmented with trial-collected ICU cost data for a further 61 participants (35 control and 26 intervention). Combining these groups, we obtained cost data for 529 participants (261 control and 268 intervention); these included 519 (256 control and 263 intervention) and 522 (259 control and 263 intervention) participants for the base case economic evaluation of stewardship and clinical cure respectively. The two trial arm populations were similar, with no clear differences for the great majority of baseline characteristics. Details on trial randomisation and participant characteristics are given elsewhere [13].

## Summary of WP3 published clinical results

Results of the clinical trial are reported elsewhere [13]. An intention to treat (ITT) analysis found that the intervention group had improved antibiotic stewardship, with 205/268 (76.5%) receiving active and proportionate antibiotics at 24 h compared with 147/263 (55.9%) in the control group, translating to a 21% absolute difference in adjusted analyses (95% CI: 0.13, 0.28). For clinical cure the intervention group had 152/268 (56.7%) deemed cured of pneumonia at 14 days compared with 171/265 (64.5%) in the control group, an estimated adjusted difference of − 6% (95% CI: − 0.15, 0.02). Due to small differences in the subsets of patients used in the economic evaluation study, measures of effectiveness reported here will vary slightly to those in the main clinical paper [13].

### Resource use and costs

We estimated the cost per test of utilising the Pneumonia Panel as £196. This estimate draws on purchase prices during the study and does not necessarily reflect current prices. For other pneumonia diagnostic tests (respiratory microbiological culture and viral PCR; X-ray; CT scan) usage was similar between the arms, with no significant differences (Additional file 1: Table S1).

A breakdown of (unadjusted) costs is given in Table 1. The cost per person for the Pneumonia Panel was £198—slightly higher than the estimated cost per test due to test failures and the consequent requirement to repeat a few tests. Unadjusted average total costs for the ICU stay were lower in the intervention arm at £32,951, compared with £40,951 in the control group (difference = − £8,000; 95% CI: − £15,894, − £106). Unadjusted mean base case costs were £40,951 for the control group compared with £33,149 for the intervention group (difference = − £7,802; 95% CI: − £15,696, £92). These results are driven by differences in ICU stay (shown in Additional file 1: Table S4). The mean length of stay was 4.2 days greater in the control group, medians were similar, but the upper quartile was longer in the control group, indicating a higher proportion of longer stays. Specific components of the ICU stay had very similar mean costs across groups, including for routine microbiological culture and PCR, X-rays, CT scans, and the use of antibiotics. When we extend beyond the base case to consider total hospital inpatient stay costs (intervention arm = £75,998; control arm = £64,459), we found that the difference in mean cost between the two arms increased to − £11,539 (95% CI: − £25,295, £2,217).

**Table 1:** Unadjusted differences in mean costs (intervention minus control) with 95% confidence intervals

Cost	Control(n = 261)		Intervention (n = 268)				
	Mean (£)	SD (£)	Mean (£)	SD (£)	Diff in means (£)	95% CI (£)	
Pneumonia Panel	0	0	198	17	198	196	200
ICU (from randomisation)	40,951	53,658	32,951	36,989	− 8,000	− 15,894	− 106
Microbiological culture & viral PCR	87	46	83	41	− 4	− 11	4
Antimicrobials	634	881	631	990	− 3	− 163	157
X–ray	183	158	185	162	1	− 26	28
CT	42	82	43	82	1	− 13	15
Base case: Pneumonia panel + ICU (from randomisation)	40,951	53,658	33,149	36,989	− 7,802	− 15,696	92
Total costs: Pneumonia + all ICU stay(s) + general admission income*	75,998	82,935	64,459	59,709	− 11,539	− 25,295	2,217

Analysis utilises available data among 529 participants who had stewardship and/or clinical cure outcomes, and ICU costs available

*Diff* difference, *SD* standard deviation, *CI* confidence interval, *ICU* intensive care unit, *PCR* polymerase chain reaction

^*^Sample sizes: control = 212 and intervention = 216

### Economic evaluation of stewardship

Results of the economic analysis in relation to stewardship are given in Table 2, with further sensitivity and subgroup analyses reported in Additional file 1: Table S2. Effectiveness is measured as the difference in proportions between groups for antibiotic stewardship (patients on active and proportionate antibiotics at 24 h): positive differences indicate improved stewardship in the intervention arm. Costs are compared using the difference in means: negative differences indicate lower costs in the Intervention arm. Based on point estimates for the base case the intervention was the preferred strategy: costs were lower, and stewardship was improved. In the base case (Table 2), costs were non-significantly lower in the intervention arm (adjusted difference in means = − £7,373; 95% CI: − £14,905, £307) and stewardship significantly improved (adjusted difference in proportions = 0.20: 95% CI: 0.12, 0.28). This finding that the intervention was preferred remained robust in the various sensitivity analyses conducted, including when costs were censored at 14 days and when costs > £200,000 were excluded (i.e. excluding high-cost outliers).

**Table 2:** Economic evaluation of stewardship with sensitivity analyses

Economic analysis	Control N	Intervention N	Treatment effect (intervention vs control)		95% CI		Interpretation
Base case	256	263	Cost (£) (diff in means)	− 7,373	− 14,905	307	Intervention preferred
			Steward. OR	2.51	1.76	3.79	
			Steward. (diff in prop)	0.20	0.12	0.28	
Base case censored to 14 days	256	263	Cost (£) (diff in means)	− 1,217	− 3,322	691	Intervention preferred
			Steward. OR	2.51	1.81	3.75	
			Steward. (diff in prop)	0.20	0.13	0.28	
Base case excluding ICU stays > 200 K	250	260	Cost (£) (diff in means)	− 4,116	− 9,443	1,350	Intervention preferred
			Steward. OR	2.44	1.73	3.73	
			Steward. (diff in prop)	0.19	0.11	0.27	
Base case adjusted*	243	242	Cost (£) (diff in means)	− 7,508	− 15,167	− 57	Intervention preferred
			Steward. OR	2.53	1.75	3.92	
			Steward. (diff in prop)	0.19	0.12	0.27	
Total costs: Pneumonia Panel + whole ICU stay + spell income	208	212	Cost (£) (diff in means)	− 10,901	− 23,743	1,834	Intervention preferred
			Steward. OR	3.18	2.18	5.12	
			Steward. (diff in prop)	0.24	0.16	0.33	

All analyses adjust for site

*CI* confidence interval, *Steward*. stewardship, *OR* adjusted odds ratio comparing Pneumonia Panel to control, *Diff* difference, *ICU* intensive care unit

^*^Base case, adjusting for baseline: covid status, SOFA/pSOFA score, and other infection

**Fig. 1:**
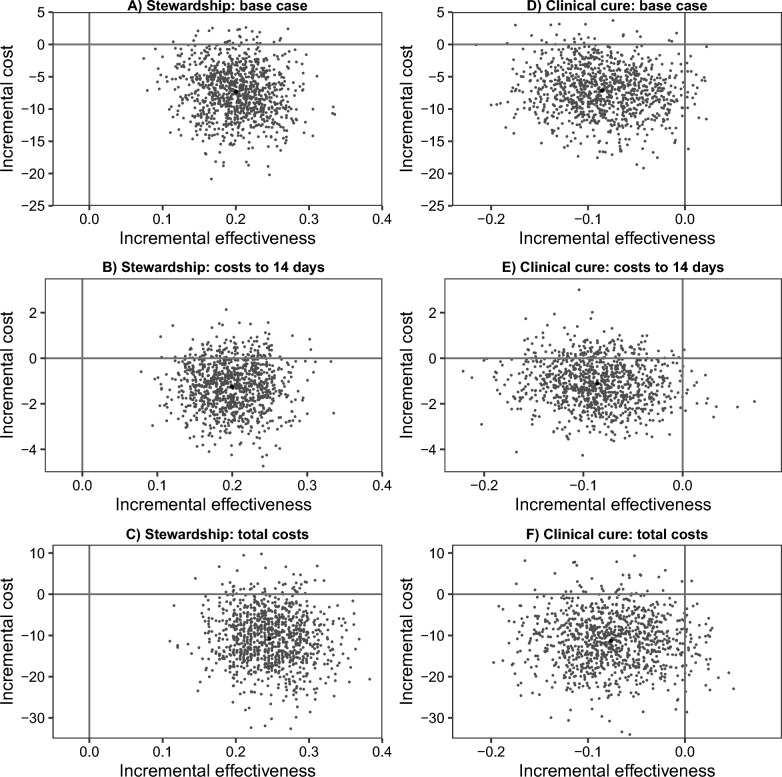
Cost–effectiveness planes for stewardship (left column) and clinical cure (right column). Cost-effectiveness (CE) planes resulting from bootstrap re-sampling for base case (first row), costs censored to 14 days (middle), and total costs (Pneumonia Panel + whole ICU stay(s) + spell income; bottom). Measures of effectiveness are the difference in proportion between arms (intervention-control). CE planes show estimates of the incremental differences (cost and proportional differences) for the intervention group compared with the control group drawn from the bootstrap resampling. These figures have horizontal and vertical axis lines shown at zero. Any estimate below the horizontal line indicates that the intervention group is cost-saving compared to the control, and any point to the right of the vertical line indicates the intervention group is more effective than the control group

**Fig. 2:**
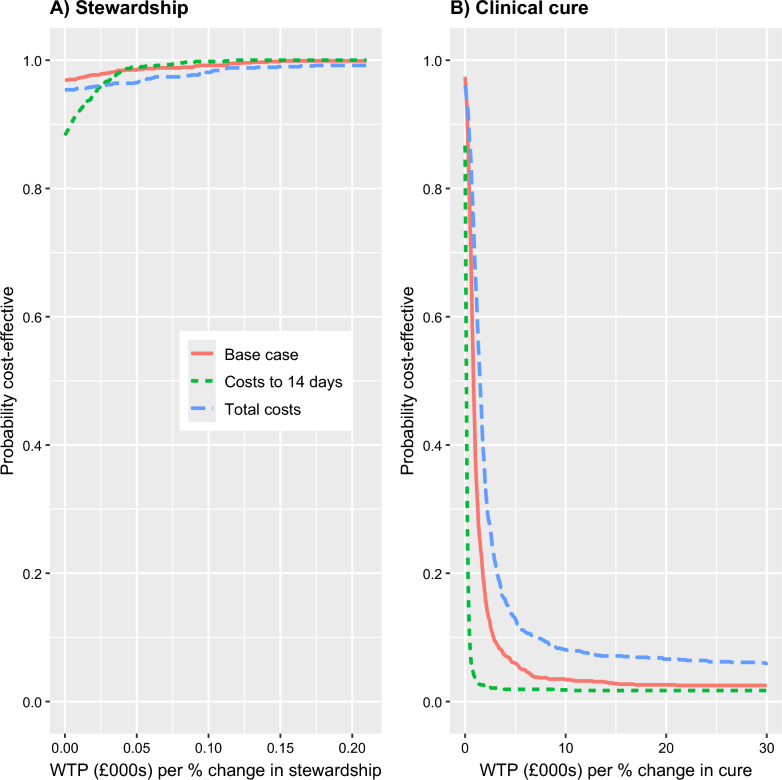
Cost–effectiveness acceptability curves for stewardship (left panel) and clinical cure (right panel)

It is also important to consider the uncertainty relating to these results and this is explored in Figs. 1 and 2 for selected analyses. Cost-effectiveness planes for stewardship are shown in the left-hand column of Fig. 1. For the base-case analyses, 97% of points of the corresponding CE plane (Fig. 1, panel A) fall in the South-East (SE) quadrant (lower costs, more effective). However, there was less certainty in this finding when costs were truncated at 14 days, with the corresponding CE plane (Fig. 1, panel B) only having 88% of points in the SE quadrant. The estimate of cost difference, favouring the intervention, increased when we considered total hospital costs (see above); however, there was increased uncertainty compared with the base case, with points on the CE place (Fig. 1, panel C) more spread out and slightly fewer points (95%) falling in the SE quadrant. Figure 2 shows cost-effectiveness acceptability curves (CEACs), these illustrate the probability that an intervention is cost-effective at different willingness to pay (WTP) for a 1% improvement in stewardship The three sensitivity analyses are shown as separate curves on the same graph (left hand panel in Fig. 2). All the analyses indicate that the intervention is highly likely to be preferred at all WTP thresholds.

Analyses by subgroup are presented in Additional file 1: Table S2. In all cases, 95% CIs were notably wide and overlap on costs and stewardship between sub-groups. Point estimates indicate greater cost reduction for children compared with adults. Point estimates also show stewardship was improved for patients with both VAP and HAP, but whereas there were cost savings for VAP patients (− £12,151; 95% CI: − £21,260, − £2,266), there was a small, non-significant increase in cost for those with HAP (£1,198; 95% CI: − £7,258, £10,692).

### Economic evaluation of clinical cure

Results of the economic analysis in relation to clinical cure are shown in Table 3, with further sensitivity and subgroup analyses in Additional file 1: Table S3. CE planes for a subset of the analyses are shown in the right-hand panels of Fig. 1. CEACs for the same subset of the analyses are shown in Fig. 2. Effectiveness was measured as the difference in proportions achieving cure between groups: negative differences indicate a lower rate of cure in the intervention arm. In nearly all conducted analyses, point estimates indicate lower costs and less effectiveness in the intervention arm. In the base case, costs were non-significantly lower in the intervention arm (− £7,147; 95% CI: − £15,198, £27) and clinical cure was significantly lower (− 0.08; 95% CI: − 0.17, − 0.003); 95% of points of the corresponding CE plane (Fig. 1, panel D) fell in the South-West (SW) quadrant (lower costs, less effective). More broadly, when compared with results for stewardship (above), the CE planes were similar in terms of the proportion of points below the horizontal, zero-cost, axis. However, the CE planes differed with most points lying to the left of the vertical axis, indicating most estimates are consistent with the intervention being less effective than in the control arm. For the CEACs (right hand panel of Fig. 2) it can be seen that there is a low probability that the intervention is cost-effective in terms of clinical cure, with the probability of cost-effectiveness falling below 0.2 across most of the WTP range considered.

**Table 3:** Economic evaluation of cure with sensitivity analyses

Economic analysis	Control N	Intervention N	Treatment effect (intervention vs control)		95% CI		Interpretation
Base case	259	263	Cost (£) (diff in means)	− 7,147	− 15,198	27	Intervention less costly, but less effective
			Cure OR	0.689	0.461	0.985	
			Cure (diff in prop)	− 0.08	− 0.17	− 0.00	
Base case censored to 14 days	259	263	Cost (£) (diff in means)	− 1,107	− 3,128	775	Intervention less costly, but less effective
			Cure OR	0.689	0.468	0.959	
			Cure (diff in prop)	− 0.08	− 0.16	− 0.01	
Base case excluding ICU stays > 200 K	253	260	Cost (£) (diff in means)	− 3,992	− 9,921	1,623	Intervention less costly, but less effective
			Cure OR	0.671	0.462	0.952	
			Cure (diff in prop)	− 0.09	− 0.17	− 0.01	
Base case adjusted*	247	241	Cost (£) (diff in means)	− 6,933	− 14,773	679	Intervention less costly, but less effective
			Cure OR	0.597	0.375	0.874	
			Cure (diff in prop)	− 0.10	− 0.17	− 0.02	
Total costs: Pneumonia Panel + whole ICU stay + spell income	210	212	Cost (£) (diff in means)	− 10,995	− 23,843	1,234	Intervention less costly, but less effective
			Cure OR	0.714	0.466	1.04	
			Cure (diff in prop)	− 0.08	− 0.16	0.01	

All analyses adjust for site

*C*I confidence interval, *OR* adjusted odds ratio comparing Pneumonia Panel to control, *ICU* intensive care unit, *Diff* difference

^*^Base case, adjusting for baseline: covid status, SOFA/pSOFA score, and other infection

## Discussion

### Summary of main findings

We found it feasible to collect resource-use data alongside clinical data in the INHALE trial and to source hospital finance data for most trial patients. The cost per test of the Pneumonia Panel was estimated at £196. We found, as expected, that the most impactful item of resource use was the cost of ICU stays, and that differences in this item far exceeded the unit cost of the Pneumonia Panel, and to a lesser extent, antimicrobial therapy costs. We found a reduction in estimated ICU costs in the intervention arm, with this difference persisting across a variety of assumptions and subgroup analyses, though differences were not always statistically significant. However, cost-effectiveness analyses produced different conclusions depending on the effectiveness measure. For stewardship, the Pneumonia Panel had a high probability of being preferred to the control, as it offered both lower costs and better stewardship. In terms of clinical cure, we did not find advantages for the intervention arm and so could not conclude an economic advantage for the Pneumonia Panel.

### Strengths of the study

The economic component took place within a well-conducted clinical trial. Hence, we collected good quality data. Data completeness was high as each participating trust engaged well with the trial team. Through trust links, we obtained finance data, allowing participants to be matched with a reliable estimate of associated trust income. We also obtained costs for the wider hospital stay, even when it extended beyond the study’s 21-day follow-up. As the study employed two co-primary outcomes, we conducted two economic evaluations focused on different aspects of treatment.

We believe this to be the first substantive economic evaluation of the Pneumonia Panel compared with standard care (empirical therapy adapted once routine microbiology results become available), specifically in the UK NHS context.

Most economic evaluations for HAP/VAP compare costs and benefits between different antibiotics, often using health economic models–for example, see Wagner et al. for a review of health economic models for HAP/VAP [20]. A number of studies have looked at the cost-effectiveness of different antibiotics [21]. However, some authors have explored alternative approaches to HAP/VAP treatment/management [22]–also approaches to prevent HAP/VAP, such as improved oral care [23]. We are only aware of one other published economic analysis of the BioFire FilmArray Pneumonia Panel, conducted in a German healthcare context: in this case, Guillotin et al*.* compared costs and the optimisation of antibiotic prescription in treating VAP [24]. The potential impact of utilising the Pneumonia Panel in the treatment of 100 patients was simulated by independent blinded experts choosing antibiotic therapies (two independent experts per case, but in cases of disagreement, six experts reviewed cases; all blinded to actual treatment) and comparing their advice with the treatment actually received. This analysis only considered costs of the test and antibiotics, finding an ICER of €1,121 (95% CI: − €7,021, €6,794) to avoid one day of non-optimized antibiotic therapy. As the exercise was, essentially, hypothetical there was no consideration of either actual prescribing or outcomes.

Obtaining data from finance departments strengthened our analysis as it enabled hospital costs beyond the 21 days of follow-up up to be captured. However, we were unable to collect this data for all participants (due to some trusts non-responding and some individuals not having been discharged at time of data gathering). We supplemented finance data with information on ICU stays up to 21 days from the study database. Supplementary Additional file 1: Tables S2 and S3 show this had a limited effect on estimated cost differences (-9,202 vs -10,712 for stewardship, and -9,249 vs -10,390 for cure).

### Limitations of study

There are limitations relating to the clinical trial design. Given the critical care setting, it was unfeasible to routinely collect patient health-related quality of life data. The design also precluded a long follow-up period. Given the severe impact that HAP/VAP can have on health, it is likely that many patients would take considerable periods to recover; consequently, the full impact on health and resources of HAP/VAP care guided by the Pneumonia Panel may not have been captured, impacting the robustness of our cost-effectiveness conclusions.

There is difficulty of interpreting the dual primary outcomes: the intervention was preferred in terms of stewardship but was less effective in terms of clinical cure. This represents a challenge to balancing the various factors of better stewardship, lower rates of clinical cure and potential cost savings. To explore these issues, we looked at ICU length of stay (Additional file 1: Table S4) and costs separated by mortality and clinical cure (Additional file 1: Tables S5 and S6). Additional file 1: Table S4 shows that the mean length of ICU stay was 4.2 days longer in the control group. Median length of stay was similar in the two groups but the upper quartile was longer for the control group, indicating that length of stay in the control group was more highly right skewed. A factor that could influence results would be differences in mortality between groups. If mortality during the ICU stay was greater in the intervention group one would expect lower ICU costs but, clearly, this would not be a desirable outcome. To investigate mortality impacts on ICU costs, we estimated arm costs split by 28-day mortality. These results are shown in Additional file 1: Table S5. We utilised data for 461 participants (224 control and 237 intervention) with both finance-department-provided costs (avoiding issues of cost-censoring) and clinical cure data. Among these patients, 30% (137/461) had died by day 28 (28%, 63/224 control; 31%, 74/237 intervention). If our findings were driven purely by mortality differences, one would expect cost differences to disappear when data was divided by mortality status. However, when considering only patients who remained alive at 28 days, we still saw higher costs in the control group. Similarly, observed differences in costs might have resulted from differences in cure rates. Accordingly, Additional file 1: Table S6 shows results divided by 14-day cure status. Again, if cost differences were driven by cure rate differences, one would expect them to narrow when divided by cure status. Nevertheless, cost differences persisted in this analysis (Additional file 1: Table S6); further, it appears that the biggest cost difference was for those individuals not cured at 14 days, where costs were substantially higher in the control group (though non-significantly, with a mean difference of − £16,182 and 95% CI: − £33,221, £858).

Lastly, as with many economic evaluations, it will be difficult to generalise findings to other health care systems, which (for example) have different funding structures and costs, as well as considerable variation in antimicrobial resistance rates, which may greatly co-determine the clinical and cost-effectiveness of a rapid diagnostic [13].

## Conclusion

INHALE WP3 found lower estimates of costs for the management of HAP/VAP using the Pneumonia Panel rather than standard care. This was not contingent on more early deaths among patients in the intervention arm. There was evidence of the Pneumonia Panel being cost-effective in terms of stewardship, but not for clinical cure; consequently, we cannot conclude an economic advantage for the pneumonia panel.

## Supplementary Information

**Additional file 1**. Supplementary Tables and Sensitivity Analyses.

## Data Availability

The data dictionary and de-identified patient data analysed and presented in this study are available from NCTU following publication, on reasonable request and subject to appropriate data sharing agreements. The health economics analysis plan is publicly available at https://norwichcrtu.uea.ac.uk/ctudocs_public/inhale/heap_1_4.pdf.

## Abbreviations

CEAC: Cost-effectiveness acceptability curve
CE plane: Cost-effectiveness plane
CI: Confidence interval
CT: Computed tomography
HAP: Hospital-acquired pneumonia
ICER: Incremental cost-effectiveness ratio
ICU: Intensive care unit
IQR: Interquartile range
ITT: Intention to treat
NHS: National Health Service
NICE: National Institute for Health and Care Excellence
OR: Odds ratio
PCR: Polymerase chain reaction
pSOFA: Paediatric Sequential Organ Failure Assessment
RCT: Randomised controlled trial
SOFA: Sequential Organ Failure Assessment
SE: South-East
SW: South-West
VAP: Ventilator-associated pneumonia
