# Correction: Cost-effectiveness of rapid, ICU-based, syndromic PCR in hospital-acquired pneumonia: analysis of the INHALE WP3 multi-centre RCT

**DOI:** 10.1186/s13054-025-05645-8

**Published:** 2025-09-16

**Authors:** Adam P. Wagner, Virve I. Enne, Vanya Gant, Susan Stirling, Julie A. Barber, David M. Livermore, David A. Turner, the INHALE WP3 study group, Naseem Ahmed, Olugbenga Akinugbe, Zoran Aman, Angela Aramburo, Georgia Bercades, David Brealey, Rhian Bull, Jane Cassidy, Jaime Carungcong, Benny P. Cherian, Antony Colles, Patricia Correia da Costa, Jeronimo Cuesta, Zaneeta Dhesi, Kerry Dresser, Matthew Dryden, Helder Filipe, Minnie Gellamucho, Ingrid Hass, Juliet High, Robert Horne, Hala Kandil, Michael Karlikowski, Nigel Klein, Kamal Liyanage, Damian Mack, Philip Milner, Sara Garcia Mingo, Daniel Martin, Luke Moore, Nabeela Mughal, Mark de Neef, Julie North, Justin O’Grady, Lauran O’Neill, Valerie Page, Alyssa Pandolfo, Robert Parker, Nehal Patel, Pooja Patel, Mark Peters, Giovanni Pipi, Karen Reid, Federico Ricciardi, Peter Riley, Charlotte Russell, Laura Shallcross, David Shaw, Suveer Singh, Ruan Simpson, Julian Sonksen, Sarah-Jane Stewart, Deborah Smyth, Ann Marie Swart, Jenny Tan, Carly Tooke, Laura Tous, Eleanor Tudtud, Ian Turner-Bone, Justin Wang, Victoria Waugh, Ingeborg D. Welters, Laura Wilding, Helen Winmill, Karen Williams, Xiaobei Zhao

**Affiliations:** 1 Faculty of Medicine and Health Sciences, Norwich Medical School, University of East Anglia, Norwich Research Park, Norwich, NR4 7TJ UK; 2 Applied Research Collaboration East of England (NIHR ARC EoE, National Institute for Health and Care Research (NIHR), Cambridgeshire and Peterborough NHS Foundation Trust, Norwich, UK; 3 Centre for Clinical Microbiology, University College London, London, UK; 4 Norwich Clinical Trials Unit, University of East Anglia, London, UK; 5 Department of Statistical Science, University College London, London, UK; 6 Department of Microbiology, University College London Hospitals, London, UK

**Correction: Critical Care (2025) 29: 352**

 10.1186/s13054-025-05428-1

Following publication of the original article [1], the authors identified an error in the author name of Virve I. Enne.

The incorrect author name is: Virvel Enne.

The correct author name is: Virve I. Enne.

The author group has been updated above and the original article [1] has been corrected.
