# Supplementary material for: Cost-effectiveness of rapid, ICU-based, syndromic PCR in hospital-acquired pneumonia: analysis of the INHALE WP3 multi-centre RCT
Source: Crit Care. 2025 Aug 8;29:352. doi: 10.1186/s13054-025-05428-1 (PMC12333181; doi:10.1186/s13054-025-05428-1)
Supplement: Supplementary file 1 — Additional file 1. Supplementary Tables and Sensitivity Analyses. [file 13054_2025_5428_MOESM1_ESM.docx]

**Supplementary materials**

**Table S1:** Unadjusted differences in mean diagnostic use with 95% confidence intervals (utilising available data among 529 participants who had stewardship and/or clinical cure outcomes, and ICU costs available).

| Test use | Control   (n=261) | | Intervention (n=268) | | Diff in means |  |  |
| --- | --- | --- | --- | --- | --- | --- | --- |
|  | Mean | SD | Mean | SD |  | 95% CI | |
| Pneumonia Panel | 0.0 | 0.0 | 1.0 | 0.1 | 1.0 | 1.0 | 1.0 |
| Microbiological culture & viral PCR | 2.4 | 2.3 | 2.2 | 2.0 | –0.2 | –0.5 | 0.2 |
| X–ray | 4.1 | 3.5 | 4.1 | 3.6 | 0.0 | –0.6 | 0.6 |
| CT | 0.3 | 0.6 | 0.3 | 0.6 | 0.0 | –0.1 | 0.1 |

Diff= difference. SD= standard deviation. CI= confidence interval. ICU= intensive care unit. PCR= polymerase chain reaction.

**Table S2:** Further economic evaluation of stewardship with sensitivity and sub-group analyses. All analyses adjust for site. Subgroup analyses conducted on base case.

| **Economic analysis** | **Control N** | **Intervention N** | **Treatment effect (intervention vs control)** | | **95% CI** | | **Interpretation** |
| --- | --- | --- | --- | --- | --- | --- | --- |
| Base case complete case only | 223 | 237 | Cost (£)   (diff in means) | –9,202 | –17,508 | –1,185 | Intervention preferred |
|  |  |  | Steward. OR | 2.73 | 1.84 | 4.32 |  |
|  |  |  | Steward.   (diff in prop) | 0.21 | 0.13 | 0.30 |  |
| Base case reference costs | 256 | 263 | Cost (£)   (diff in means) | –10,712 | –22,994 | 969 | Intervention preferred |
|  |  |  | Steward. OR | 2.51 | 1.74 | 3.74 |  |
|  |  |  | Steward.   (diff in prop) | 0.20 | 0.12 | 0.27 |  |
| Subgroup: Adult | 219 | 222 | Cost (£)   (diff in means) | –6,874 | –14,651 | 607 | Intervention preferred |
|  |  |  | Steward. OR | 2.7 | 1.81 | 4.31 |  |
|  |  |  | Steward.   (diff in prop) | 0.21 | 0.13 | 0.30 |  |
| Subgroup: Child | 37 | 41 | Cost (£)   (diff in means) | –11,114 | –39,190 | 15,684 | Intervention preferred |
|  |  |  | Steward. OR | 1.66 | 0.583 | 4.92 |  |
|  |  |  | Steward.   (diff in prop) | 0.11 | –0.10 | 0.31 |  |
| Subgroup: Has Covid at rand | 87 | 91 | Cost (£)   (diff in means) | –6,589 | –20,489 | 5,691 | Intervention preferred |
|  |  |  | Steward. OR | 4.17 | 2.27 | 8.55 |  |
|  |  |  | Steward.   (diff in prop) | 0.32 | 0.19 | 0.46 |  |
| Subgroup: No Covid at rand | 146 | 148 | Cost (£)   (diff in means) | –6,883 | –17,524 | 2,759 | Intervention preferred |
|  |  |  | Steward. OR | 1.77 | 1.15 | 2.89 |  |
|  |  |  | Steward.   (diff in prop) | 0.12 | 0.03 | 0.21 |  |
| Subgroup: HAP | 83 | 78 | Cost (£)   (diff in means) | 1,198 | –7,258 | 10,692 | Intervention more costly, more effective |
|  |  |  | Steward. OR | 1.52 | 0.774 | 2.91 |  |
|  |  |  | Steward.   (diff in prop) | 0.09 | –0.06 | 0.23 |  |
| Subgroup: VAP | 173 | 184 | Cost (£)   (diff in means) | –12,151 | –21,260 | –2,266 | Intervention preferred |
|  |  |  | Steward. OR | 3.15 | 2.03 | 5.31 |  |
|  |  |  | Steward.   (diff in prop) | 0.24 | 0.15 | 0.34 |  |

CI= confidence interval. Steward.= stewardship. OR= adjusted odds ratio comparing Pneumonia Panel to control. ICU=intensive care unit. Diff=difference

* Base case, adjusting for baseline: covid status, SOFA/pSOFA score, and other infection.

**Table S3:** Further economic evaluation of cure with sensitivity and sub-group analyses. All analyses adjust for site. Subgroup analyses conducted on base case analysis.

| **Economic analysis** | **Control N** | **Intervention N** | **Treatment effect**   **(intervention vs control)** | | **95% CI** | | **Interpretation** |
| --- | --- | --- | --- | --- | --- | --- | --- |
| Base case complete case only | 224 | 237 | Cost (£)   (diff in means) | –9,249 | –17,646 | –435 | Intervention less costly, but less effective |
|  |  |  | Cure OR | 0.667 | 0.442 | 0.989 |  |
|  |  |  | Cure   (diff in prop) | –0.09 | –0.17 | –0.00 |  |
| Base case reference costs | 259 | 263 | Cost (£)   (diff in means) | –10,390 | –22,380 | 439 | Intervention less costly, but less effective |
|  |  |  | Cure OR | 0.689 | 0.479 | 0.981 |  |
|  |  |  | Cure   (diff in prop) | –0.08 | –0.16 | –0.00 |  |
| Subgroup: Adult | 222 | 223 | Cost (£)   (diff in means) | –6,870 | –14,353 | –225 | Intervention less costly, but less effective |
|  |  |  | Cure OR | 0.68 | 0.448 | 1.01 |  |
|  |  |  | Cure   (diff in prop) | –0.09 | –0.18 | 0.00 |  |
| Subgroup: Child | 37 | 40 | Cost (£)   (diff in means) | –9,670 | –35,691 | 18,083 | Intervention less costly, but less effective |
|  |  |  | Cure OR | 0.748 | 0.208 | 2.58 |  |
|  |  |  | Cure   (diff in prop) | –0.04 | –0.15 | 0.12 |  |
| Subgroup: Has Covid at rand | 89 | 91 | Cost (£)   (diff in means) | –5,638 | –20,254 | 6,280 | Intervention less costly, but less effective |
|  |  |  | Cure OR | 0.57 | 0.296 | 1.02 |  |
|  |  |  | Cure   (diff in prop) | –0.12 | –0.26 | 0.00 |  |
| Subgroup: No Covid at rand | 147 | 147 | Cost (£)   (diff in means) | –6,893 | –16,342 | 2,989 | Intervention less costly, but less effective |
|  |  |  | Cure OR | 0.695 | 0.405 | 1.13 |  |
|  |  |  | Cure   (diff in prop) | –0.07 | –0.16 | 0.02 |  |
| Subgroup: HAP | 83 | 79 | Cost (£)   (diff in means) | 1,034 | –7,648 | 10,304 | Control preferred |
|  |  |  | Cure OR | 0.758 | 0.391 | 1.58 |  |
|  |  |  | Cure   (diff in prop) | –0.05 | –0.17 | 0.08 |  |
| Subgroup: VAP | 176 | 184 | Cost (£)   (diff in means) | –11,477 | –21,134 | –2,432 | Intervention less costly, but less effective |
|  |  |  | Cure OR | 0.668 | 0.422 | 1.02 |  |
|  |  |  | Cure   (diff in prop) | –0.09 | –0.19 | 0.00 |  |

CI= confidence interval. OR= adjusted odds ratio comparing pneumonia panel to control. ICU=intensive care unit. Diff=difference

* Base case, adjusting for baseline: covid status, SOFA/pSOFA score, and other infection.

**Table S4:** ICU length of stay (LOS; days) from randomisation to ICU exit (death or discharge) compared using: i) means, standard deviation and t-test; ii) median, interquartile range and quantile regression. Restricted to participants with finance department provided cost data and clinical cure outcome.

|  | **Control (N=224)** |  | **Intervention (N=237)** |  |  |  |
| --- | --- | --- | --- | --- | --- | --- |
| **ICU LOS from randomisation** | **Mean/Median** | **SD/IQR** | **Mean/Median** | **SD/IQR** | Diff. | **95%CI** |
| Mean/SD/T-test | 22.5 | 28.5 | 18.4 | 18.9 | -4.2 | (-8.6 to 0.3) |
| Median/IQR/ Quantile regression | 13 | (7 to 26) | 12 | (6 to 23) | -1 | (-8.3 to 2.3) |

**Table S5:** Unadjusted differences in mean ICU costs from randomisation to ICU exit (death or discharge) with 95% confidence intervals, overall and by mortality by 28 days. Restricted to participants with finance-department provided cost data and clinical cure outcome.

|  | Control | | | Intervention | | |  |  |  |
| --- | --- | --- | --- | --- | --- | --- | --- | --- | --- |
| ICU costs | N | Mean (£) | SD (£) | N | Mean (£) | SD (£) | Mean dif (£) | 95% CI (£) | |
| All | 224 | 44,763 | 56,878 | 237 | 34,746 | 38,839 | –10,017 | –18,985 | –1,049 |
| Alive at, or discharged by, 28 days | 161 | 54,161 | 63,820 | 163 | 43,016 | 43,337 | –11,145 | –23,090 | 799 |
| Died by 28 days | 63 | 20,747 | 17,593 | 74 | 16,531 | 14,831 | –4,216 | –9,775 | 1,343 |

*ICU= intensive care unit. SD= standard deviation. CI= confidence interval. dif= difference.*

**Table S6:** Unadjusted differences in mean ICU costs from randomisation to ICU exit (death or discharge) with 95% confidence intervals, overall and by clinical cure at 14 days. Restricted to participants with finance-department provided cost data and clinical cure outcome.

|  | Control | | | Intervention | | |  |  |  |
| --- | --- | --- | --- | --- | --- | --- | --- | --- | --- |
| ICU costs | N | Mean (£) | SD (£) | N | Mean (£) | SD (£) | Mean dif (£) | 95% CI (£) | |
| All | 224 | 44,763 | 56,878 | 237 | 34,746 | 38,839 | –10,017 | –18,985 | –1,049 |
| Not cured | 82 | 53,513 | 68,633 | 107 | 37,332 | 42,273 | –16,182 | –33,221 | 858 |
| Cured | 142 | 39,711 | 48,376 | 130 | 32,619 | 35,795 | –7,092 | –17,198 | 3,013 |

ICU= intensive care unit. SD= standard deviation. CI= confidence interval. dif= difference.
